# A reproducible dynamic phantom for sequence testing in hyperpolarised ^13^C-magnetic resonance

**DOI:** 10.1259/bjr.20210770

**Published:** 2022-03-08

**Authors:** Rafat Chowdhury, Marianthi-Vasiliki Papoutsaki, Christoph A Müller, Lorna Smith, Fiona Gong, Max Bullock, Harriet Rogers, Manju Mathew, Tom Syer, Saurabh Singh, Adam Retter, Lucy Caselton, Jung Ryu, Aaron Oliver-Taylor, Xavier Golay, Alan Bainbridge, David G Gadian, Shonit Punwani

**Affiliations:** Centre for Medical Imaging, Division of Medicine, University College London, London, UK; Centre for Medical Imaging, Division of Medicine, University College London, London, UK; Department of Radiology, Medical Physics, Medical Center – University of Freiburg, Faculty of Medicine, University of Freiburg, Freiburg, Germany; German Cancer Consortium (DKTK), partner site Freiburg, German Cancer Research Center (DKFZ), Heidelberg, Germany; Gold Standard Phantoms Limited, London, UK; Centre for Medical Imaging, Division of Medicine, University College London, London, UK; Centre for Medical Imaging, Division of Medicine, University College London, London, UK; Centre for Medical Imaging, Division of Medicine, University College London, London, UK; Centre for Medical Imaging, Division of Medicine, University College London, London, UK; Centre for Medical Imaging, Division of Medicine, University College London, London, UK; Centre for Medical Imaging, Division of Medicine, University College London, London, UK; Centre for Medical Imaging, Division of Medicine, University College London, London, UK; Centre for Medical Imaging, Division of Medicine, University College London, London, UK; Centre for Medical Imaging, Division of Medicine, University College London, London, UK; Gold Standard Phantoms Limited, London, UK; Gold Standard Phantoms Limited, London, UK; Department of Brain Repair and Rehabilitation, Institute of Neurology, Queen’s Square, University College London, London, UK; Department of Medical Physics and Biomedical Engineering, University College London Hospitals, London, UK; UCL Great Ormond Street Institute of Child Health, London, UK; Centre for Medical Imaging, Division of Medicine, University College London, London, UK; Department of Radiology, University College London Hospitals NHS Foundation Trust, London, UK

## Abstract

**Objective:**

To develop a phantom system which can be integrated with an automated injection system, eliminating the experimental variability that arises with manual injection; for the purposes of pulse sequence testing and metric derivation in hyperpolarised ^13^C-MR.

**Methods:**

The custom dynamic phantom was machined from Ultem and filled with a nicotinamide adenine dinucleotide and lactate dehydrogenase mixture dissolved in phosphate buffered saline. Hyperpolarised [1-^13^C]-pyruvate was then injected into the phantom (*n* = 8) via an automated syringe pump and the conversion of pyruvate to lactate monitored through a ^13^C imaging sequence.

**Results:**

The phantom showed low coefficient of variation for the lactate to pyruvate peak signal heights (11.6%) and dynamic area-under curve ratios (11.0%). The variance for the lactate dehydrogenase enzyme rate constant (kP) was also seen to be low at 15.6%.

**Conclusion:**

The dynamic phantom demonstrates high reproducibility for quantification of ^13^C-hyperpolarised MR-derived metrics. Establishing such a phantom is needed to facilitate development of hyperpolarsed ^13^C-MR pulse sequenced; and moreover, to enable multisite hyperpolarised ^13^C-MR clinical trials where assessment of metric variability across sites is critical.

**Advances in knowledge:**

The dynamic phantom developed during the course of this study will be a useful tool in testing new pulse sequences and standardisation in future hyperpolarised work.

## Introduction

**Figure 1.**
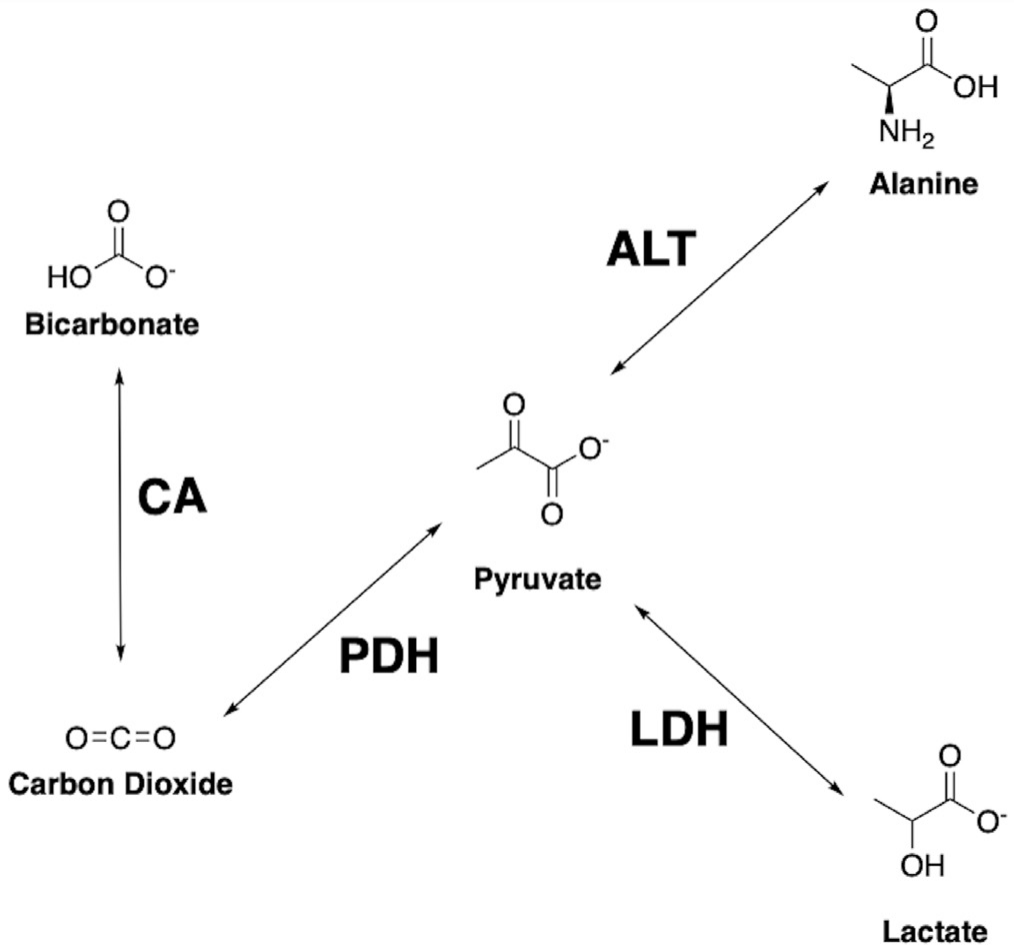
Important downstream metabolites from pyruvate and their respective enzyme pathways. Pyruvate can be converted into lactate via LDH, alanine through ALT, carbon dioxide by PDH and subsequently bicarbonate produced from CA. ALT, alanine aminotransferase; CA, carbonic anhydrase; LDH, lactate dehydrogenase; PDH, pyruvate dehydrogenase

The use of hyperpolarised [1-^13^C] pyruvate, in conjunction with ^13^C-Magnetic Resonance Spectroscopy/Imaging (^13^C-MR), has been demonstrated as a promising technique in allowing for the non-invasive assessment of metabolic processes in real-time.^1,2^ There has been a particular focus on the preferred conversion of pyruvate into lactate (Figure 1), despite the presence of oxygen, specifically in tumorous tissue.^1–3^ Recent studies have aimed to use hyperpolarised ^13^C-MR (HYP-MR) to delineate tumour aggressiveness by attempting to correlate metabolic activity with existing classification techniques.^4^

The short-lived hyperpolarised state, combined with the cost of a clinical hyperpolarised study, however, necessitates the need for optimised pulse sequences, with high spectral, spatial and temporal resolutions.^3,5,6^ Moreover, a consensus has yet to be achieved regarding the best metric to quantify the data obtained during hyperpolarised studies, with some studies demonstrate the use of kinetic models,^7^ whilst others have favoured model-free approaches.^4^

Sequences optimised in a preclinical setting require additional testing prior to clinical use. Whilst animal models have been preferred in the past,^5,6^ the reproducibility of in vitro models has been demonstrated in recent work.^8^ Such models, referred to as dynamic phantoms, due to the varying nature of the signal they produce as a function of time, have also been used in preclinical studies to compare different analysis methods.^9^

Dynamic phantoms, in principle, follow a common experimental pathway. Firstly, the phantom is filled with a buffer and an enzyme, which varies depending on the reaction being observed. The hyperpolarised agent, such as [1-^13^C] pyruvate or otherwise, is then introduced to the system and mixed, with data acquisition then beginning through ^13^C-MR spectroscopy or imaging.^8–11^

The primary limitation of existing dynamic phantoms is a lack of automated injection and mixing systems. Previous studies required removing dynamic phantoms from MR scanners or manual injection and mixing within the scanner. The former resulted in a portion of the metabolic process being missed.^9^ The latter comes with magnetic field inhomogeneities, introduced via physical movement, potentially harming the acquisition.^11^

Here, we propose a dynamic phantom compatible with an automated injection system and describe its overall reproducibility over *n* = 8 HYP-MR experiments.

## Methods and materials

The reproducibility study was performed on a Siemens Biograph mMR 3T system (Siemens Healthineers, Erlangen, Germany), using a custom-designed ^13^C clamshell transmit and dual tuned ^1^H/^13^C endorectal, receive-only coil (RAPID Biomedical GmbH, Rimpar, Germany), alongside a MEDRAD Spectris Solaris EP MR injection system (MEDRAD, Pennsylvania). All chemicals used in this study were sourced from Merck (Merck KGaA, Darmstadt, Germany); Merck Life Science UK Limited): L-LDH from rabbit muscle (SKU 10127876001), 5 ml; β-nicotinamide adenine dinucleotide(NADH), reduced disodium salt hydrate (SKU N8129), 1 g; phosphate buffered saline (PBS), pH 7.2, (SKU 806544), 1 L.

Data processing was performed using MATLAB (Mathworks, Massachusetts). Each metabolite signal time course was normalised to its maximum pyruvate signal, allowing for different data sets (*n* = 8) to be compared. Quantification of the time courses was performed using some previously described methods.^9^ These are further explained in the Supplementary Material 1 (Figure S2).

### Phantom design

**Figure 2.**
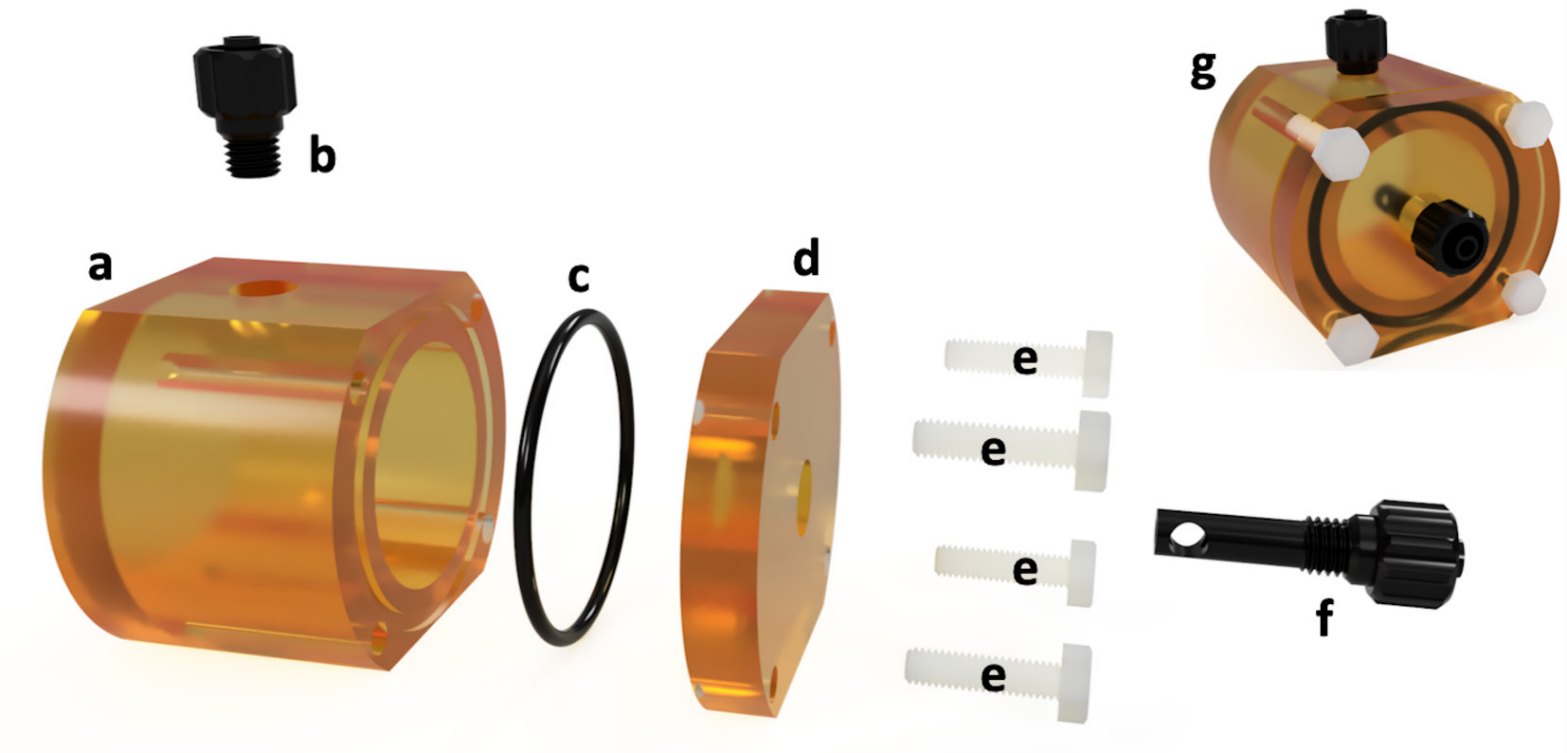
Visualisation of the dynamic phantom, created and rendered in Autodesk’s Fusion 360 – (a) main body of the phantom, with a hole for (b) a ¼” threaded male Luer lock outlet and a groove for (c) a nitrile O-ring. (**d**) The lid is fixed to the main body via (e) four 6,6-Nylon M3 screws, whilst an additional hole was added to the lid to allow for (f) a custom inlet to be fitted.

A phantom, machined from ULTEM 1000^TM^, was produced (Figure 2) and specifically designed to work in combination with a clinical pump injection system through the use of standardised Luer connectors at the phantoms inlet (2f) and outlet (2b). A distribution mechanism (2f) was incorporated in the form of a channel that disperses contrast agent at the inner chambers geometric centre.

The phantom (Figure 2) is composed of three main components: the body (a), an O-ring (c), and the lid (d). The body possesses a chamber with an internal volume of 25 ml, with a Luer lock (b) attached acting as a pressure outlet during hyperpolarised agent delivery. The lid was designed with a Luer lock acting as an inlet (f) with nylon screws (e) affixing the lid to the body. The inlet and outlet ports (Cole-Parmer Instrument Company, Cambridgeshire) are both male Luer lock to threaded adapters, ¼” internal diameter times ¼” national pipe thread (M). A 5.17 mm external diameter by 20 mm length polypropylene tube was attached to the threaded end of the inlet port. A 3.5 mm internal diameter by 13 mm length hole was drilled into the tube. A 4.5 mm diameter hole was created 2 mm from the end of the tube perpendicular to the inner bore. The use of a nitrile O-ring ensured the phantom was leak-proof. Technical specifications are available in the supplementary materials (Figure 1). The cleaning process requires the removal of the screws, affording access to the entire main compartment. The inlet and outlet are threaded and, as such, can be unscrewed and removed for further cleaning if necessary.

The modular design and ease of disassembly allow for the phantom to be readily reused, affording the repeated use of a system with the capacity for remote and automated injection. During experiments the phantom was connected to a MEDRAD Spectris Solaris EP MR injection system using 65/115 ml MRI Syringe Sets. The endorectal coil was fitted underneath the phantom using a stand (Rapid Biomedical GmbH, Rimpar, Germany). Reference markers on both the ^13^C clamshell transmit and endorectal coil were used to confirm the alignment of the phantom prior to each experiment.

### Enzyme buffer

Prior to the injection of hyperpolarised [1-^13^C] pyruvate, the dynamic phantom was filled with a mixture of LDH (16 µg ml^−1^) and NADH (4.4 mM) in PBS (equal to 153 U). After filling, the dynamic phantom was connected to the power injector through an injection line (7 ml dead volume) filled with buffered saline. Strict timings were imposed with the injection of hyperpolarised [1-^13^C] pyruvate occurring within 20 min of the buffer being prepared to avoid any potential enzyme denaturation or cofactor decay.^12^

### Hyperpolarised [1-^13^C] pyruvate

A GE SPINLab Hyperpolariser was used to produce the hyperpolarised [1-^13^C] pyruvate. [1-^13^C] pyruvic acid was mixed with AH111501 electron paramagnetic agent (EPA), loaded into a fluid path and placed in the Hyperpolariser, as previously described.^1^ The acid and EPA mixture was cooled to <1K, producing a uniform glass, and irradiated with microwaves for approximately 2 h, achieving a polarisation level of 25.6±3.2% (*n* = 8). Prior to injection, the hyperpolarised glass was dissolved with sterile water (38 ml) and neutralised with 17.5 g sterile trometamol buffer (333 mM Tris and 600 mM NaOH), affording liquid state, hyperpolarised [1-^13^C] pyruvate (250 mM, 39.5 ± 1.1 ml, pH 7.20 ± 0.29). The syringe holding the [1-^13^C] pyruvate solution was then loaded into the power injector connected to the dynamic phantom. Data acquisition began at the start of injection. A total of 4 ml of hyperpolarised [1-^13^C] pyruvate was delivered to the phantom (1 ml s^−1^).

### ^1^H-imaging

The dynamic phantom was placed on top of an endorectal coil, with 1.5 L water bottles placed on either side for coil loading. A turbo spin-echo sequence was utilised, in the sagittal and axial planes, to align the dynamic phantom with the most sensitive part of the endorectal coil, indicated by a [^13^C] urea phantom within the coil: field of view (FOV): 180 × 140 × 90 mm^3^, voxel size: 0.7 × 0.7 × 3 mm^3^, slice thickness: 3 mm, no. of slices: 30, repetition time (TR): 5400 ms, echo time (TE): 109 ms, echo train length (ETL): 15, no. of signal averages (NSA): 1, excitation flip angle (FA): 90°. Additionally, a dual gradient echo sequence was obtained for each experiment; FOV: 360 × 360 × 80 mm^3^, voxel size: 1.4 × 1.4 × 10 mm^3^, FA: 15°, TR: 329 ms, TE_1_: 2.39 ms, TE_2_: 7.17 ms, ETL: 2, NSA: 3. The latter was used to calculate a field map used to process the ^13^C images for metabolite map reconstruction via a previously described Iterative Decomposition of water and fat with Echo Asymmetry and Least-square estimation (IDEAL) model.^11^

### ^13^C-MR (metabolic imaging)

A 3D multiecho balanced steady-state free precession (ME-bSSFP) sequence was used for ^13^C-MR acquisition: FOV: 90 × 90×80 mm^3^, voxel size: 11.3 × 11.3×10 mm^3^, FA: 24°, TR: 15.8 ms, no. of bipolar gradient echoes per TR (NE): 7, ΔTE: 1.1 ms, NSA: 6, scan time (TA): 6.2 s. The transmit and receive frequencies were centred on the lactate resonant frequency (Δf_Lac_ = 0 Hz, chemical shift: 185 ppm), based on the resonance frequency from the urea in the [^13^C] urea phantom (chemical shift: 165 ppm) inside the endorectal coil. A gradient bandwidth of BW_read_ = 1200 Hz/px was utilised in the multiecho read-out to minimise alternating chemical shift displacements of the off-resonant metabolites in the bipolar read-out. Three consecutive ME-bSSFP acquisitions were conducted directly after the completion of hyperpolarised [1-^13^C] pyruvate injection, followed by the acquisition of a non-localised spectrum using a single non-localised free induction decay (FID) sequence: FA: 10°, TR: 1000 ms, NSA: 1, BW: 4000 Hz. After the FID, a further three ME-bSSFP acquisitions and another non-localised spectrum followed, and the scan list was repeated in this manner for a total acquisition time of 2 min 30 s.

## Results

**Figure 3.**
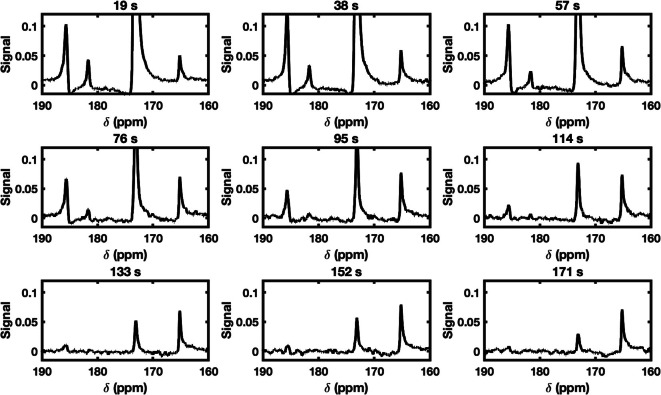
Change in the metabolite signals observed in the non-localised spectra after the injection of hyperpolarised [1-^13^C] pyruvate into the dynamic phantom. The spectra were normalised to the strongest signal ([1-^13^C] pyruvate at 172 ppm) observed in the first spectrum (19 s). The spectra are zoomed in upon to show all the signals - [^13^C] Urea (165 ppm), [1-^13^C] pyruvate (172 ppm), [1-^13^C] pyruvate hydrate (181 ppm) and [1-^13^C] lactate (185 ppm) signals.

**Figure 4.**
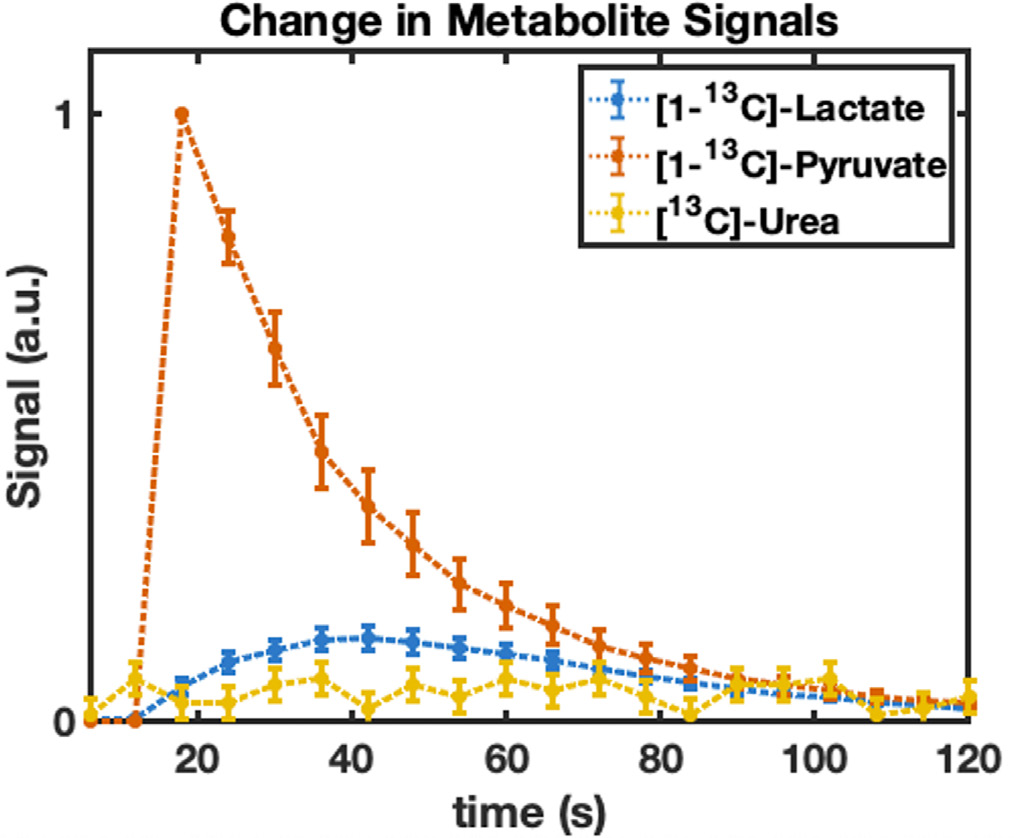
Change in [1-^13^C] lactate (blue) and [1-^13^C] pyruvate (red) signals, within the dynamic phantom, over time, across all HYP-MR experiments (*n* = 8). The signal from the [^13^C] urea reference phantom (yellow) within the endorectal coil is also shown.

**Figure 5.**
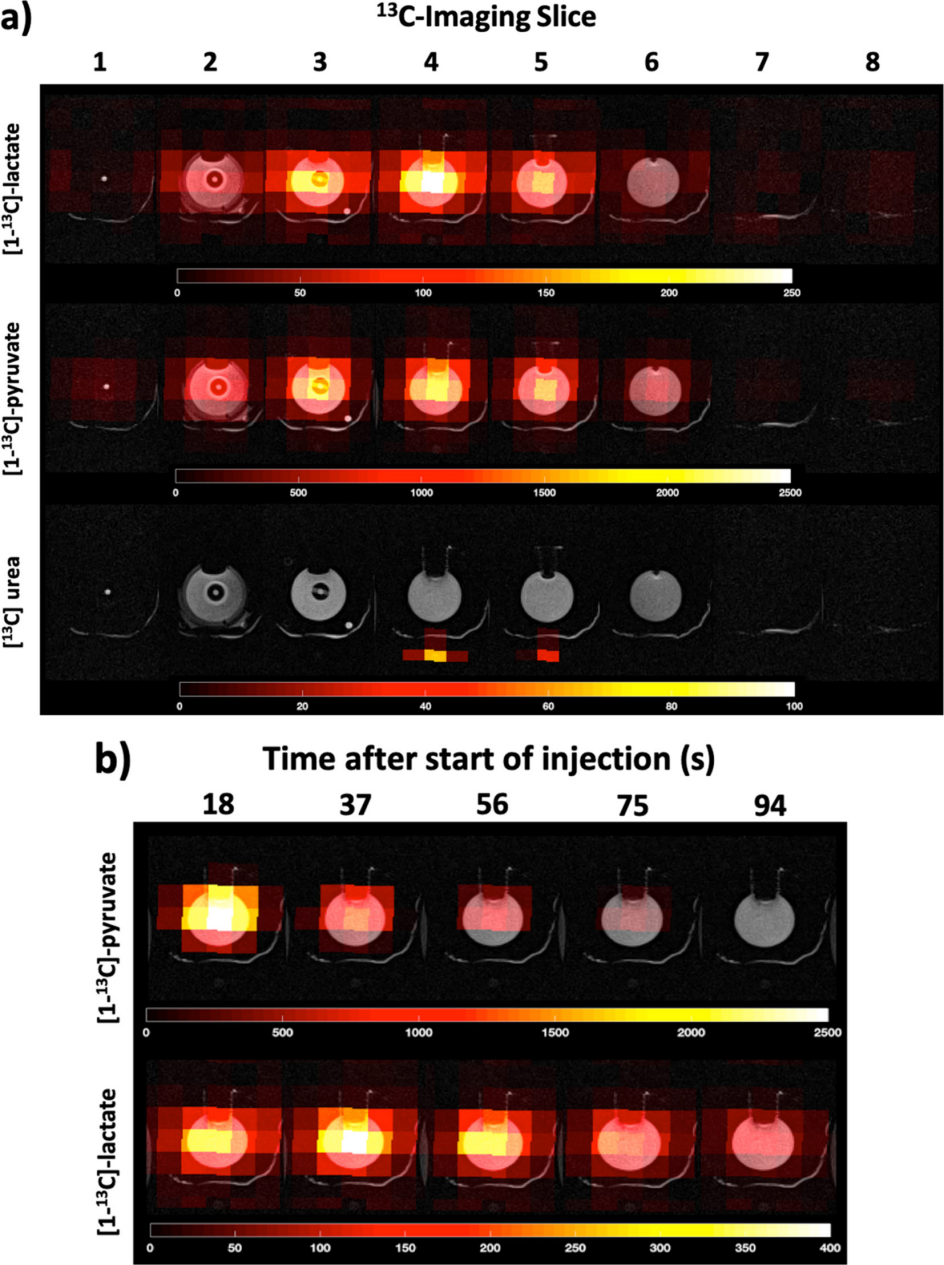
(**a**) Distribution of [1-^13^C] pyruvate and [1-^13^C] lactate within the dynamic phantom, at *t* = 12 s are shown. The metabolite maps were overlaid on a set of *T*_2_ weighted reference images. The [^13^C] urea signal from within the endorectal receive coil is also shown. (**b**) The change in [1-^13^C] pyruvate and [1-^13^C] lactate signals at the centre of the phantom are shown up to 94 s after the start of injection. Note, that slice no. 4 is shown in( b) with a slice thickness 10 mm. It is likely that this slice includes parts of the two injection nozzles which divide the pyruvate injection to the left and right part of the chamber, shown by the increased intensities to left and right of the chamber. The images are scaled to the maximum signal intensity (arbitrary units) of pyruvate and lactate in their respective time courses.

The non-localised spectra (Figure 3) showed signals at the chemical shifts: 165, 172, 181 and 185 ppm; representative of [^13^C] urea, [1-^13^C] pyruvate, [1-^13^C] pyruvate hydrate and [1-^13^C] lactate, respectively. The magnitude of the urea signal was constant, as expected, across all time points, due to its coming only from the static phantom inside the endorectal coil itself. Both non-localised spectra (Figure 3) and analysis of ^13^C-imaging (Figure 4), show the dynamic [1-^13^C] pyruvate signal peaked at approximately 18–19 s, with the [1-^13^C] lactate appearing to peak at approximately 37 s (Figure 4). Both [1-^13^C] pyruvate and [1-^13^C] lactate decayed due to T_1_ relaxation but were still visible in the spectra 133 s after the start of injection. [1-^13^C] pyruvate hydrate was also observed, with its peak disappearing 76 s after the start of injection (Figure 3). The ME-bSSFP metabolite maps (Figure 5) localise both signals, [1-^13^C] lactate and [1-^13^C] pyruvate, within the dynamic phantom. The [1-^13^C] lactate signals showed a slow build-up and decay curve, while the [1-^13^C] pyruvate signal showed fast arrival in the chamber followed by decay. Quantification of these images was performed by plotting the change in individual metabolite signals for the voxels covering the entire phantom (Figure 4). Analysis of the time curves (Table 1) was performed using some previously described methods,^9^ which are detailed in the Supplementary Material 1.

**Table 1.** Analysis of data obtained across all experiments through previously described methods^8^)

Method	Mean	Standard deviation	Coefficient of variation (%)
**Lac-Pyr peak ratio**	0.27	0.03	11.6
**Lac-Pyr AUC ratio**	0.19	0.02	11.0
**k_P_ (s^−1^**)	0.041	0.006	15.5
**T_1_ pyruvate**	31	7	23

AUC, area under the curve.

The change in metabolite signals from the ^13^C-imaging data were quantified to gauge the reproducibility of the dynamic phantom. [1-^13^C] lactate to [1-^13^C] pyruvate peak and area under the curve ratios (Supplementary Material 1-Figure S2) were found to have the lowest coefficient of variation across all measurements at 11.6% and 11%, respectively.

## Discussion

The dynamic phantom demonstrates the capacity to observe the conversion of [1-^13^C] pyruvate, through LDH, into [1-^13^C] lactate (Figures 1 and 5), in a reaction analogous to that observed in clinical HYP-MR studies.^1,2^ The phantom has allowed us to capture the entire enzymatic reaction time course, with remote injection eliminating the need to remove the device from the MR scanner, as seen in previous studies.^9^ The dynamic phantom’s large size and use of a pressure outlet also eliminated the need to mix the relevant enzyme and hyperpolarised contrast prior to injection in the phantom.^8^

Three factors can contribute to the rate of decline in [1-^13^C] pyruvate signal (Figures 3–5): relaxation of the hyperpolarised state, deterioration of the signal via radiofrequency pulse excitation, and lastly, enzymatic conversion via LDH into [1-^13^C] lactate. The evolution of the [1-^13^C] lactate signal reflects its active enzymatic production from [1-^13^C] pyruvate, which then, similarly to [1-^13^C] pyruvate, undergoes an active signal decay due to both relaxation and pulse excitation.

The metabolite signal time courses (Figure 4) offer a complete visualisation of the chemical conversion of [1-^13^C] pyruvate to [1-^13^C] lactate when compared to previous dynamic phantoms,^9^ as the current system allows for the entire process of [1-^13^C] lactate signal generation being captured. Previous designs required mixing of the hyperpolarised agent and enzyme solution outside of the phantom, prior to injection, causing a portion of the reaction to be missed. Notably, the coefficient of variation (*n* = 8) for the metabolite signals at each time point was calculated as <20% and <14% for [1-^13^C] pyruvate and [1-^13^C] lactate, respectively (Figure 4), demonstrating the reproducibility of the experiments using the dynamic phantom.

The analysis of the metabolite signal time courses demonstrates how such a phantom can be used to investigate a variety of metrics of interest (Table 1). The analysis methods demonstrate the robust and reproducible nature of the dynamic phantom (Table 1). The area under the curve (AUC) method, which has been shown, under certain conditions, to provide a model-free surrogate of k_p (7,13)_, showed the lowest coefficient of variance across all analytical methods, possibly due to its insensitivity to pyruvate uptake, unlike established kinetic models.^9^ In clinical HYP-MR automated injection of the hyperpolarised agent is a standardised practice to reduce variability. Factors such as bolus arrival and flow rate can influence these metrics, however the design presented allows for the precise control of these parameters which is reflected in the reproducibility observed in Figure S3. This phantom takes our HYP-MR experiments one step closer to the clinical reality.

The non-localised spectra (Figure 3) indicate the presence of [1-^13^C] pyruvate hydrate (181 ppm). Pyruvate hydrate is not metabolic active but is in very rapid exchange in equilibrium with pyruvate. At pH 7.5–8.2, the ratio of pyruvate hydrate to pyruvate is approximately 8% or smaller with enhanced formation of pyruvate hydrate at lower pH.^13–15^ The experimental setup used and the use of PBS buffer, meant that pH throughout our experiments was in the physiological range and approximately constant. As such, after the initial formation of the pyruvate hydrate the equilibrium was unperturbed, and the [1-^13^C] pyruvate hydrate signal undergoes the typical decay we see with hyperpolarised species. The [1-^13^C] lactate signal, however, is formed due to the interaction of pyruvate with the LDH enzyme; this reaction continues until the contribution of signal decay of the hyperpolarised state exceeds the gain due to the enzymatic reaction, causing the signal curves to initially rise and then drop off, as seen in Figure 4. Notably, the spectra provide no information for spatial discrimination and are primarily affected by the coil’s sensitivity profile.

Enzymatic reactions are temperature sensitive, to mitigate the impact of temperature on the experiments the LDH and NADH were allowed to equilibrate to room temperature prior to preparing the phantom solution. There is also variation in the polarisation of the pyruvate upon dissolution (between 25.0 and 37.0°C).^16^ This alongside the difficulty in measuring the temperature of the phantom solution post-injection is a limitation of the system. Despite this, the metrics extracted across the experiments undertaken in this body of work and the coefficients of variance (11.6% for the lactate to pyruvate AUC ratio) calculated suggest that the system has a good experimental reproducibility.

Prior to both clinical and preclinical HYP-MR studies, MR scanners most often need to be elaborately calibrated and adjusted for quality assurance purposes.^11,17^ This study utilised an endorectal coil with an in-built [^13^C] urea phantom. It is uncommon, unfortunately, to find other multinuclear coil types with similarly in-built phantoms for reference. This could be solved in the current design by adding a static chamber, filled with ^13^C labelled compound, within or on top of the current dynamic phantom system, allowing for calibration and adjustment without changing the experimental setup.

Another factor to consider involves the amount of hyperpolarised [1-^13^C] pyruvate used in comparison to how much is needed for a single experiment. Typically, for clinical experiments, 40 ml of hyperpolarised [1-^13^C] pyruvate are produced per fluid path, with each taking >4 h to produce. Adjusting the formulation used to prepare hyperpolarisation fluid paths is possible.

The primary purpose of amending the formulation of the hyperpolarised pyruvate solution would be as a cost-saving measure through the use of a smaller volume of the very expensive PA/EPA mix. The experiments in this study, using the MEDRAD 65/115 ml MRI Syringe Sets requiring 11 ml of hyperpolarised pyruvate, whilst in each instance approx. 40 ml was produced. This required the use of 1.35 g of EPA/PA mixture every time. Scaling the amount of EPA/PA by a factor of 3.2–0.4219 g and reducing the volume of neutralisation and dissolution media accordingly will produce approximately 12.5 ml of hyperpolarised solution, minimising the amount of unused solution. Another possible development may also involve integrating multiple chambers into the current design for simultaneous experiments with different concentrations of enzymes. Such dynamic phantoms with multiple chambers have been described previously^11,18^ but did not have the capacity for automated injection.

## Summary

In this work, we have illustrated a design for a dynamic phantom system for in vitro hyperpolarised metabolic imaging experiments. The system demonstrably reduced the variance of kinetic analysis outcomes by incorporating an automated injection system for the hyperpolarised agent. The capacity for integrating any automated injection system found in a scanner room and the means to vary enzyme concentration within the phantom indicate that this setup has the opportunity to mimic a range of metabolic reactions, at varying rates, potentially reflecting different tissue types. This system could also be used as part of more extensive studies, identifying the best metric by which to quantify dynamic, hyperpolarised, ^13^C metabolic imaging data; or supporting the development and optimisation of new ^13^C-MR sequences for possible future clinical routine. Beyond this, there is also scope to quantify and derive the boundaries for tumorous and healthy tissue within the context of an optimal metric.

## Supplementary Material

bjr.20210770.suppl-01
