# Supplementary material for: A reproducible dynamic phantom for sequence testing in hyperpolarised 13C-magnetic resonance
Source: Br J Radiol. 2022 Mar 8;95(1134):20210770. doi: 10.1259/bjr.20210770 (PMC10996405; doi:10.1259/bjr.20210770)
Supplement: bjr.20210770.suppl-01 [file bjr.20210770.suppl-01.docx]

**Supplementary information**

**Schematic design for Dynamic phantom**

**
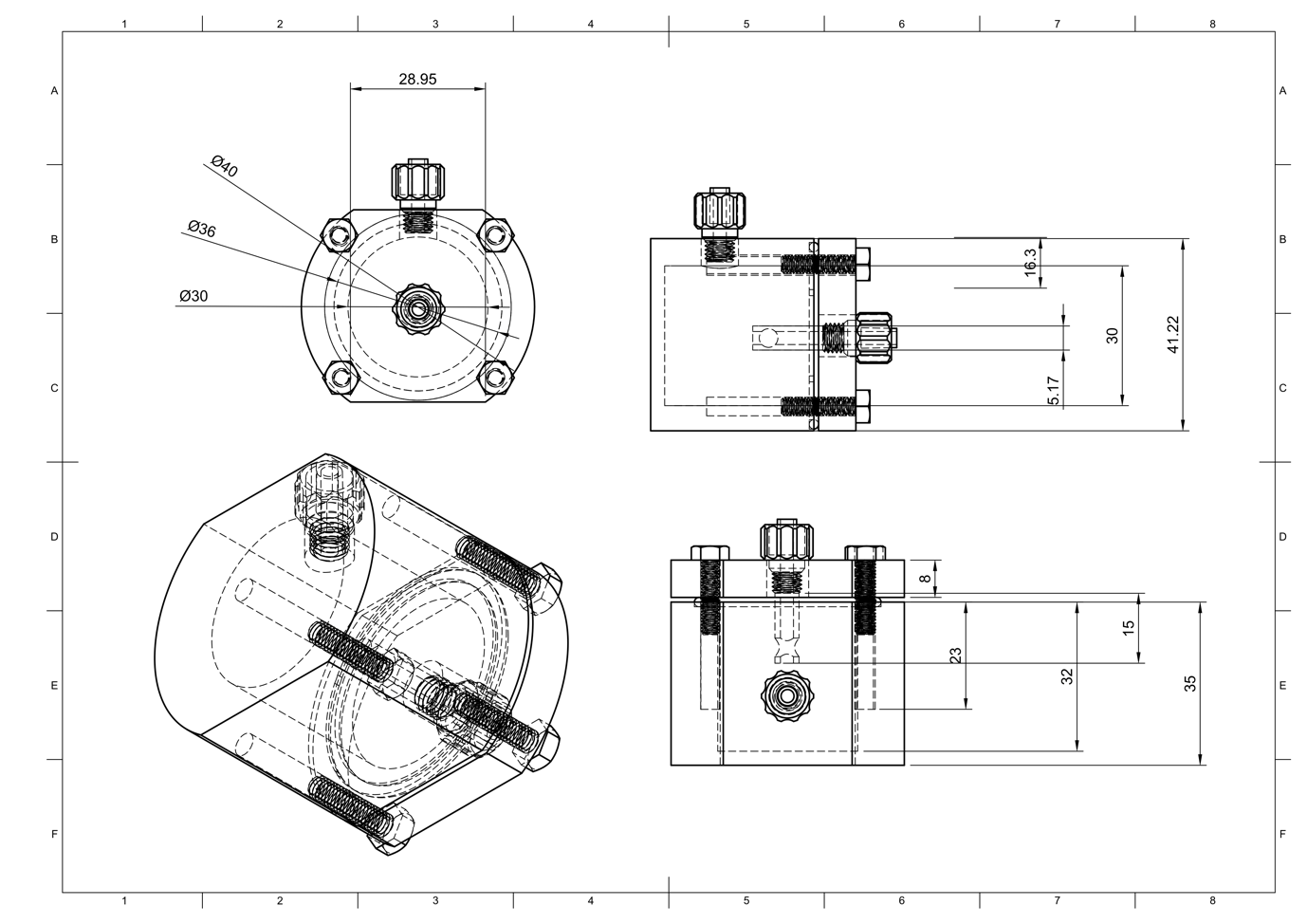
**

Figure S 1 - Schematic indicating the exact dimensions of the components within the dynamic phantom

**Analysis methods**


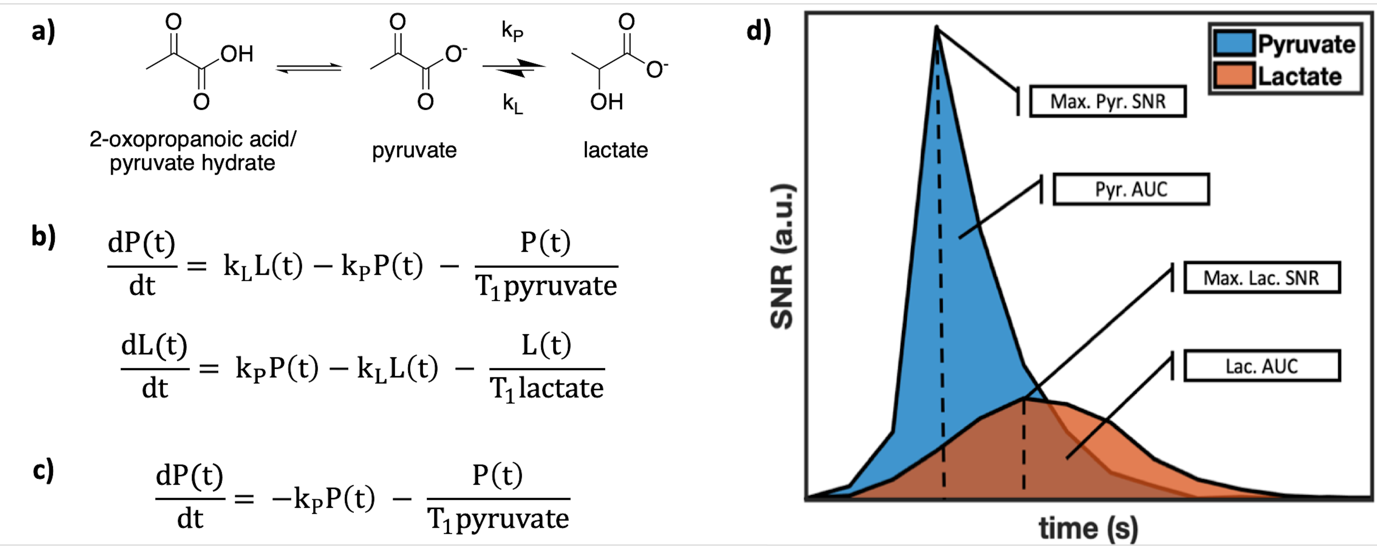


Figure S 2 - a) Chemical reactions that [1-^13^C] pyruvate undergoes in this series of experiments. Pyruvate hydrate and pyruvate are in a pH-dependent equilibrium, whilst pyruvate is then enzymatically converted into lactate via lactate dehydrogenase. Importantly, the rate of lactate generation is several orders of magnitude greater than the production of pyruvate hydrate. The consumption of pyruvate induces an equilibrium imbalance resulting in the formation of pyruvate from pyruvate hydrate. b) The differential equations for the enzymatic conversion of pyruvate to lactate, which describe the forward (top) and reverse (bottom) reactions respectively in terms of rate constants k_P_ (forward) and k_L_ (reverse). c) These equations were adapted to eliminate the reverse reaction in the fitting process by assuming k_L_ = 0, producing a one-directional kinetic model from which k_P_ and the T_1_ of pyruvate were calculated. d) The graphical methods used for the analysis of the metabolite signal time curves in this study. The ratio of the lactate to pyruvate signals when lactate is at a maximum was calculated. The second graphical metric involved deriving the ratio of the area un the curve ratio of lactate to pyruvate.

**Metabolite signal time courses**

**
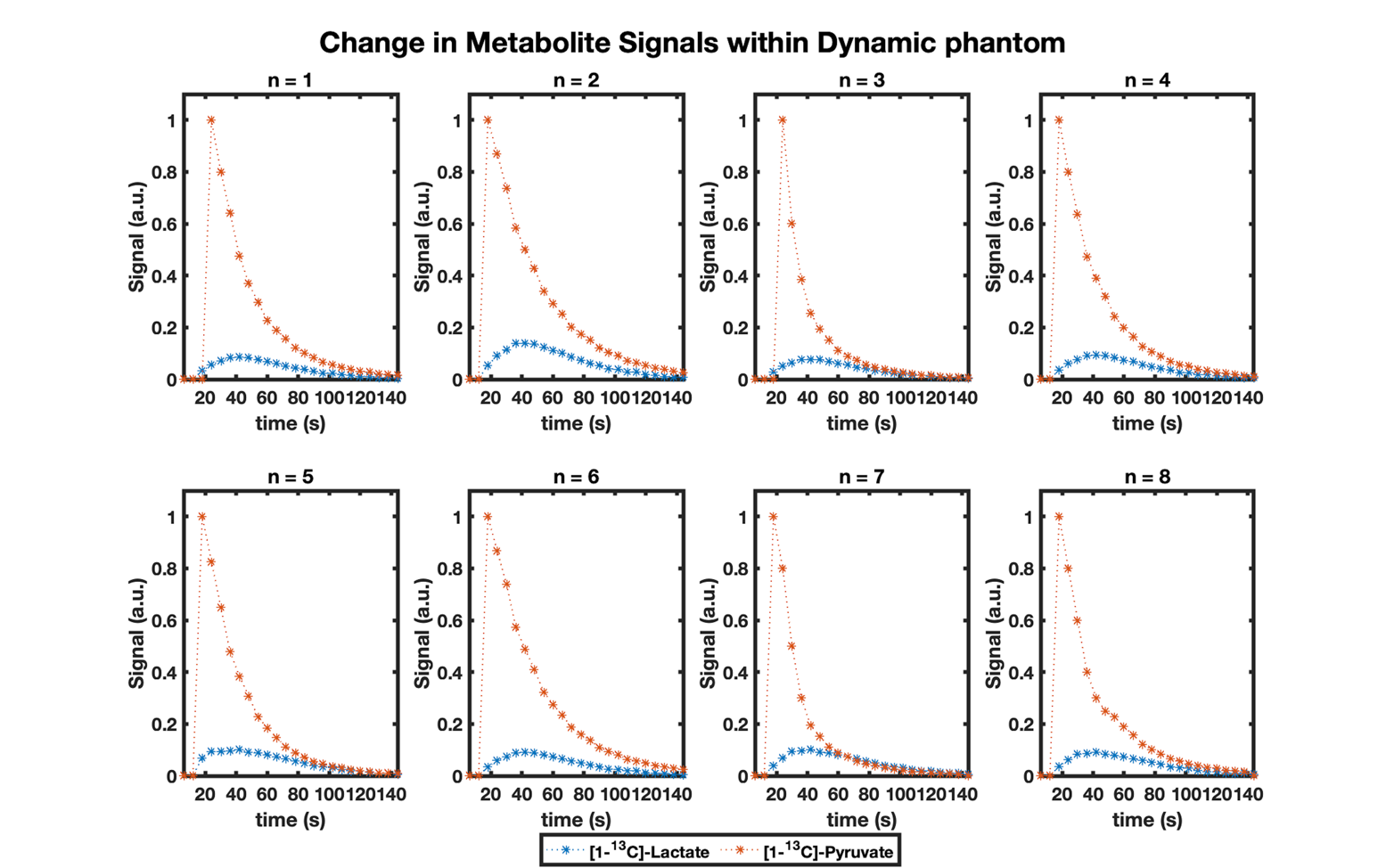
**

Figure S 3 – The change in [1-^13^C] lactate (blue) and [1-^13^C] pyruvate (red) signals within the dynamic phantom up to 2m30s after the start of injection of 4ml hyperpolarised [1-^13^C] pyruvate. Time curves are shown for all eight runs in the reproducibility study. These time curves were used to produce the analyses below.

| **Experiment no.** | **Graphical methods** | | **One-way kinetic model kL ≠ 0** | |
| --- | --- | --- | --- | --- |
|  | **Lactate to pyruvate peak ratio (a.u.)** | **Lactate to pyruvate AUC ratio (a.u.)** | **kP (s^-1^)** | **T1eff (s)** |
| **1** | 0.28 | 0.18 | 0.034 | 27 |
| **2** | 0.28 | 0.20 | 0.046 | 32 |
| **3** | 0.31 | 0.22 | 0.051 | 40 |
| **4** | 0.24 | 0.19 | 0.040 | 24 |
| **5** | 0.26 | 0.22 | 0.044 | 22 |
| **6** | 0.24 | 0.15 | 0.031 | 40 |
| **7** | 0.25 | 0.21 | 0.040 | 37 |
| **8** | 0.32 | 0.20 | 0.041 | 26 |
| **Mean** | 0.27 | 0.19 | 0.041 | 31 |
| **Standard deviation** | 0.03 | 0.02 | 0.006 | 7 |
| **Coefficient of variation (%)** | 11.62 | 10.97 | 15.45 | 23 |

Table S 1 - Graphical and kinetic analysis was performed on the metabolite time curves from all experiments in this study, using the methods outlined in Figure S1.
